# Phenotypic characterization of virological failure following lopinavir/ritonavir monotherapy using full-length *gag–protease* genes

**DOI:** 10.1093/jac/dku296

**Published:** 2014-08-04

**Authors:** Katherine A. Sutherland, Jean L. Mbisa, Jade Ghosn, Marie-Laure Chaix, Isabelle Cohen-Codar, Stephane Hue, Jean-Francois Delfraissy, Constance Delaugerre, Ravindra K. Gupta

**Affiliations:** 1 Public Health England, London, UK; 2 Université Paris Descartes, EA 7327, Faculté de Médecine Site Necker, Paris, France; 3 APHP, UF de Thérapeutique en Immuno Infectiologie, CHU Hotel Dieu, Paris, France; 4 AbbVie Laboratory, Rungis, France; 5 Division of Infection and Immunity, University College London, London, UK; 6 AP-HP, Department of Internal Medicine, Bicetre University Hospital, Le Kremlin-Bicetre, France; 7 Virology, U941 INSERM Paris Diderot University, St Louis Hospital-APHP, Paris, France

**Keywords:** HIV, protease inhibitors, Gag, monotherapy, antiretroviral resistance

## Abstract

**Objectives:**

Major protease mutations are rarely observed following first-line failure with PIs and interpretation of genotyping results in this context may be difficult. We performed extensive phenotyping of viruses from five patients failing lopinavir/ritonavir monotherapy in the MONARK study without major PI mutations by standard genotyping.

**Methods:**

Phenotypic susceptibility testing and viral infectivity assessments were performed using a single-cycle assay and fold changes (FC) relative to a lopinavir-susceptible reference strain were calculated.

**Results:**

>10-fold reduced baseline susceptibility to lopinavir occurred in two of five patients and >5-fold in another two. Four of five patients exhibited phylogenetic evidence of a limited viral evolution between baseline and failure, with amino acid changes at drug resistance-associated positions in one: T81A emerged in Gag with M36I in the protease gene, correlating with a reduction in lopinavir susceptibility from FC 7 (95% CI 6–8.35) to FC 13 (95% CI 8.11–17.8). Reductions in darunavir susceptibility (>5 FC) occurred in three individuals.

**Discussion:**

This study suggests both baseline reduced susceptibility and evolution of resistance could be contributing factors to PI failure, despite the absence of classical PI resistance mutations by standard testing methods. Use of phenotyping also reveals lower darunavir susceptibility, warranting further study as this agent is commonly used following lopinavir failure.

## Introduction

Global scale-up of antiretroviral therapy is witnessing a transition of substantial numbers of patients to second-line regimens containing the PI lopinavir coformulated with the booster agent ritonavir (lopinavir/ritonavir). Given the high prevalence of multiple NRTI mutations at virological failure in resource-limited settings (conferring cross-resistance to other NRTI),^1–5^ there has been concern that second-line treatment may result in functional monotherapy in some individuals. Indeed, rates of virological failure to PI-based second-line therapies are significant.^6–8^ In these settings, lopinavir/ritonavir monotherapy has been explored in patients failing the WHO-recommended first-line therapy with a non-NRTI regimen and found to be inferior to triple-drug regimens containing a PI.^9–12^

In better-resourced settings, only one trial to date has explored first-line ritonavir-boosted PI monotherapy (PI/r; in the form of lopinavir/ritonavir) in antiretroviral-naive patients.^13,14^ In contrast, PI/r monotherapy has been explored as a maintenance regimen in multiple trials as a strategy to reduce cost and risk of toxicity over the longer term.^15–17^ The MONARK trial investigated the efficacy of lopinavir/ritonavir monotherapy in comparison with lopinavir/ritonavir + two NRTIs at treatment initiation in treatment-naive patients. Higher rates of virological failure were reported in the monotherapy arm.^13,14^ Of the 23 genotyped patients experiencing virological failure in the lopinavir/ritonavir monotherapy arm at 96 weeks, 5 (21.7%) had major PI resistance mutations versus 0/5 of those genotyped in the triple-therapy arm.^18^ These data are consistent with a wide literature on virological failure with PI/r, where lower prevalence of major PI resistance mutations relative to other classes is generally observed.^19,20^ An earlier analysis of MONARK exploring non-protease determinants of failure had reported that pre-therapy mutations in Gag cleavage site sequences were significantly associated with the virological outcome of a first-line lopinavir/ritonavir single-drug regimen, in spite of the absence of consistent association with either the emergence of major PI resistance mutations or with changes in Gag sequences at the time of virological failure.^21^

Numerous studies have provided evidence for the role of Gag in PI susceptibility, with amino acid changes located both in and outside of the Gag cleavage sites (reviewed in Fun *et al.*^22^). Identification of broad genotypic predictors of PI susceptibility using Gag–protease remains elusive, most probably due to the importance of coevolution of residues on individual Gag sequences.^23,24^ The inclusion of full-length patient-derived Gag alongside its coevolved protease in *in vitro* phenotypic assays has been shown to substantially affect PI susceptibility (as compared with the use of protease sequences alone) in patients not exposed to PI^25,26^ and in a heavily treated patient with multiple major resistance mutations in protease.^27^ There are no data using this method for the most relevant clinical application: assessment of susceptibility to PI following failure of this class of drug (when major mutations in protease are not detected by standard population sequencing-based methods).

Given the clinical and public health needs to better understand virological failure on PI therapy, and the lack of predictive power of *gag–protease* genotyping, this study sought to phenotypically investigate treatment failure using multiple coevolved, full-length *gag–protease* sequences from patient plasma at baseline and time of treatment failure on PI monotherapy. We specifically wished to determine whether viral failure in the absence of major PI mutations (by population sequencing) was accompanied by reductions in phenotypic susceptibility to lopinavir and the other PIs saquinavir, atazanavir and darunavir.

## Methods

### Amplification of full-length *gag–protease* from study participants

**Figure 1.**
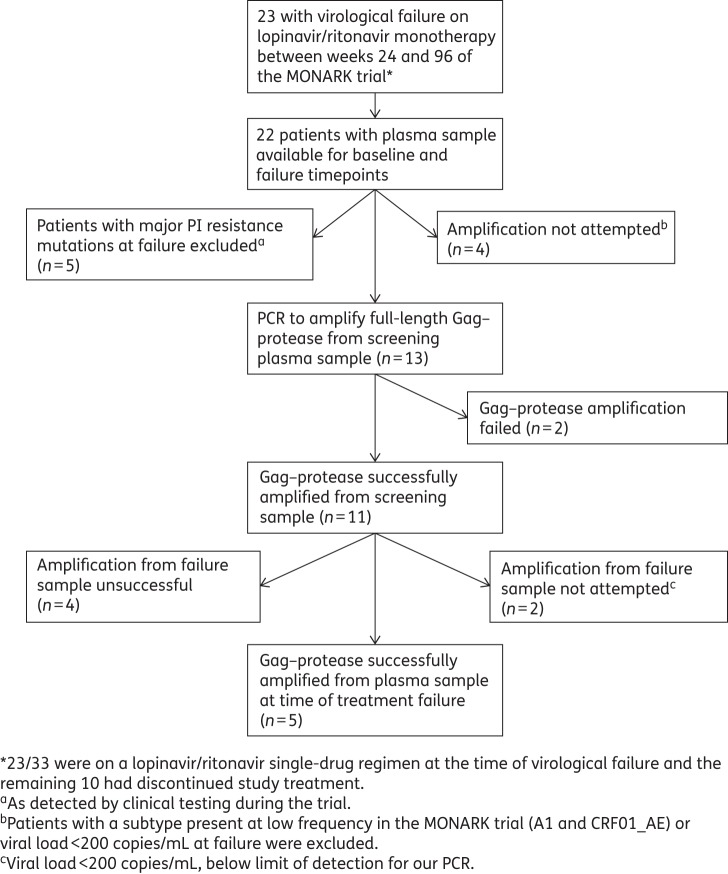
Patient sample selection flow diagram.

Paired baseline and failure samples of the 23 patients experiencing virological failure whilst on lopinavir/ritonavir drug selective pressure in MONARK (up to week 96) were available. Full-length *gag–protease* from both baseline and failure plasma samples was successfully amplified from five patients. Reasons for exclusion of the other 18 patients are presented in Figure 1.

Full-length *gag–protease* was amplified from patient samples as previously described.^26,28^ Clonal sequence analysis of 10 viral variants for each sample was performed in MEGA 5.0 software using ClustalW. The variant that most closely represented the consensus for each timepoint was selected for phenotypic testing, along with other variants of interest.

### PI susceptibility testing

PI susceptibility and single-round infectious titre were determined using previously described single-cycle assays.^25,27^ In this assay, normalization by p24 or volume of viral input dose have been shown to be equivalent for multiple viruses.^27,29^

### Phylogenetics

Sequences were aligned in MEGA 5.0 software^30^ using the ClustalW algorithm and imported into the PHYLIP program for phylogeny construction (http://evolution.genetics.washington.edu/phylip.html) using the maximum likelihood method under the generalized time reversible (GTR) model of nucleotide substitution.^31^ Maximum likelihood trees were constructed with the confidence tested using 500 bootstrap replications. Phylogenetic trees were viewed using FigTree v1.3.1 (http://tree.bio.ed.ac.uk/software/figtree/) and MEGA 5.0 software.

### Mean pairwise genetic distance (MPWGD) calculation

Genetic distance calculation was carried out in MEGA 4.1 using default parameters, based on amino acid alignments. Data were expressed as mean amino acid substitutions per site. Differences in MPWGD between screening and failure were calculated using Student's *t*-test (GraphPad Prism, La Jolla, CA, USA).

## Results

### Study group characteristics

The samples from five patients underwent sequence analysis as described in Figure 1. Clinical and virological information for these five patients including viral load, subtype and time of treatment failure is shown in Table 1. Patients randomized to the lopinavir/ritonavir monotherapy arm harboured the following HIV subtypes: 68% B, 16% CRF02_AG, 2% A, 4% G and 10% other subtypes.^18^ The five patients detailed here were infected with subtypes B (two patients), CRF02_AG (two patients) and G (one patient), as determined by the REGA subtyping tool^32,33^ (http://dbpartners.stanford.edu:8080/RegaSubtyping/stanford-hiv/typingtool/). The patients had a range of viral loads at time of failure (Table 1). In addition, the five patients experienced virological failure from week 24 (failure to achieve the primary endpoint of viral load <400 copies/mL by week 24) to week 96 (the total length of trial follow-up). Therapy adherence data from compliance assessments carried out at nine timepoints and lopinavir trough concentrations measured in the blood plasma at three timepoints were available.

**Table 1.** Clinical data on participants

Patient	Subtype	Screening viral load (copies/mL)	Failure time (weeks)	Failure viral load (copies/mL)
1403	CRF02_AG	44 600	24	24 000
3204	B	23 800	40	603
1404	CRF02_AG	166 000	48	212
4201	G	79 500	48	342
508	B	37 800	96	25 300

### Reduced baseline susceptibility with further reduction at failure

One individual (Patient 1403) was infected with a subtype CRF02_AG virus and experienced therapy failure at week 24 with a viral load at 24 000 copies/mL. At this timepoint, very low non-suppressive lopinavir levels were present in the plasma (75 ng/mL) and missed doses were reported. Clonal sequence analysis revealed the presence of the V82A major PI resistance mutation in 1 of the 10 viral variants isolated from the time of treatment failure, as well as a number of protease polymorphisms at both screening and failure (Table S1, available as Supplementary data at *JAC* Online). In addition, a number of mutations previously associated with PI susceptibility or resistance in Gag were present at both screening and time of treatment failure: E12K, R76K, T375N, I376V and L449P (Table S2).^22^ The emergence of Y79F in failure variant 6 (F6) and F9 correlated with PI exposure.

**Figure 2.**
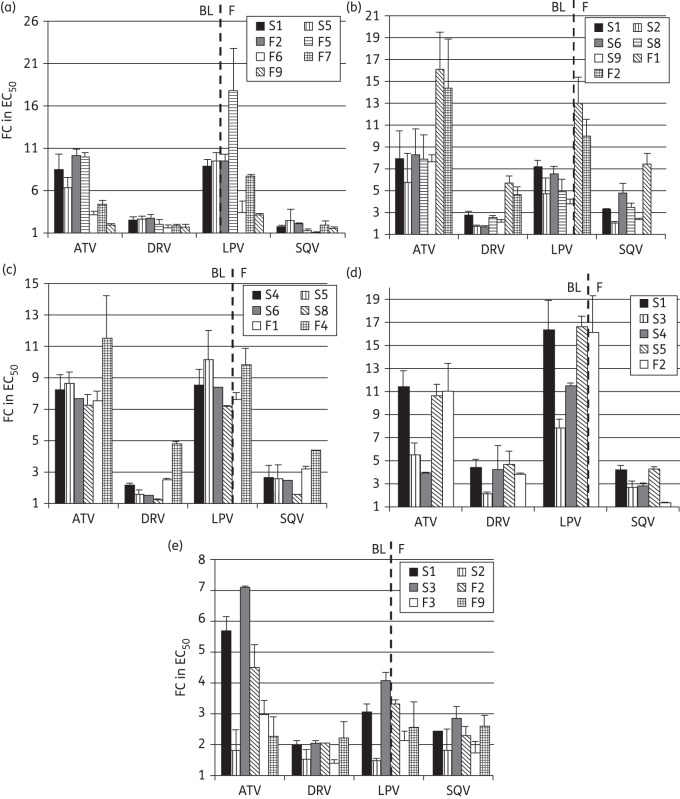
Phenotypic PI susceptibility. Phenotypic susceptibility to four PIs atazanavir (ATV), darunavir (DRV), lopinavir (LPV) and saquinavir (SQV) was determined for viral variants derived from five patients: (a) 1403, (b) 3204, (c) 1404, (d) 4201 and (e) 508. Data are presented as FC in EC_50_ in comparison with the assay reference strain. BL, baseline; F, failure.

**Figure 3.**
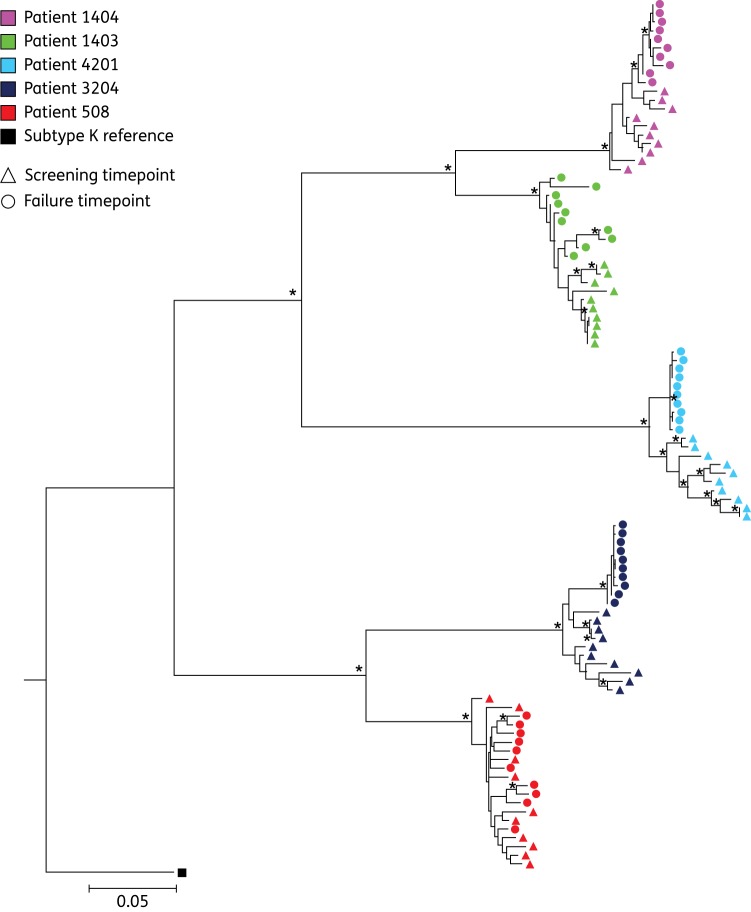
Maximum likelihood phylogeny. A maximum likelihood tree constructed using the GTR model in PhyML using an alignment of all variants from baseline and failure timepoints from five patients. Variants from each patient are represented by a single colour, the screening variants by triangles and those from failure with circles. Nodes supported by >75% bootstrapping (≥350/500) are marked by an asterisk (*). This figure appears in colour in the online version of *JAC* and in black and white in the print version of *JAC*.

Phenotypic drug susceptibility and infectivity was tested for seven variants from Patient 1403: two from the time of screening (S1 and S5) and five from the time of treatment failure (F2, F5, F6, F7 and F9). Of note, the F5 variant containing the major PI resistance mutation V82A displayed significantly reduced susceptibility to lopinavir (17-fold). For the remaining variants, reduced susceptibility in comparison with the reference strain was observed to both atazanavir and lopinavir [>10-fold change (FC) in EC_50_] for S1, F2 and F5. In contrast, F6 and F9 displayed susceptibilities similar to the assay reference strain to atazanavir and lopinavir, despite emergence of Y79F (Figure 2a). Susceptibility levels similar to that of the reference strain were observed for all variants to the PIs darunavir and saquinavir and use of EC_90_ instead of EC_50_ to calculate FCs did not significantly change the relationships between clones, although the magnitude of the FCs was reduced (Figure S1). There was little variation in MPWGD at screening and time of failure (0.007 versus 0.009 nucleotide substitutions per site, *P* = 0.0765) and screening and failure viral variants were phylogenetically distinct by maximum likelihood analysis (Figure 3).

The second individual (Patient 3204) was infected with a subtype B virus and experienced virological failure at week 40 (Table 1). No lopinavir trough concentration was available at this timepoint; however, at weeks 4 and 24, lopinavir trough concentrations were >3000 ng/mL and no missed doses were reported. A number of polymorphisms were present in protease at both screening and failure: E35D, L63P, I72V, V77I and I93L (Table S1). In addition, a number of mutations that have been previously associated with PI susceptibility or resistance in Gag were present at both screening and time of treatment failure: Y79F, T81A, M200I, H219Q, S373P, T375N, R380K, I389T and S451N (Table S2). A number of amino acid positions correlated with PI exposure: 12, 63, 70, 310, 370 and 376 in Gag and 36 in protease (Tables S1 and S2). T81A was seen in only 1 of 10 screening variants but in all failure variants. Overall, E12K, T81A and M36I were of greatest interest as they have been previously associated with PI exposure and reduced susceptibility.^34,35^

Seven viral variants were tested phenotypically, five from baseline (S1, S2, S6, S8 and S9) and two from the time of treatment failure (F1 and F2). Figure 2(b) shows that screening variants had modestly reduced susceptibility (∼7 FC) to lopinavir, atazanavir and saquinavir. Failure variants displayed lower susceptibilities to atazanavir, darunavir, lopinavir and saquinavir than the screening variants. This was most pronounced for atazanavir and lopinavir, where up to a 16-fold and 13-fold reduction in susceptibility was observed in comparison with the assay reference strain, respectively (Figure 2b). Surprisingly, moderately reduced susceptibility was also observed for darunavir, of up to 5 FC for the failure viral variants. Use of EC_90_ did not significantly change the relationships between clones (Figure S1). There was a significant difference in MPWGD between baseline (0.019) and time of treatment failure (0.001, *P* < 0.0001) and phylogeny was consistent with ongoing evolution and selection during PI therapy (Figure 3).

### Reduced baseline phenotypic susceptibility with no change at failure

Patient 1404 was infected with subtype CRF02_AG virus and experienced virological failure at week 48 of the trial with a low viral load of 212 copies/mL. The lopinavir plasma trough concentration was unavailable for this timepoint, although the lopinavir level at week 24 was <1000 ng/mL and missed doses were reported at weeks 20, 40 and 48. The minor resistance mutation A71T was present in protease of viral variants from screening and the time of treatment failure, along with a number of polymorphisms: I13A, K20I, M36I, R41K, H69K, L89I and I93M (Table S1). In addition, a number of mutations that have been previously associated with PI susceptibility or resistance in Gag were present at both screening and time of treatment failure: E12K, R76K, V370A and L449P (Table S2).

Six viral variants in total were cloned into the resistance test vector and underwent phenotypic analysis: four from baseline (S4, S5, S6 and S8) and two from the failure timepoint (F1 and F4). For this patient, reduced PI susceptibility to atazanavir and lopinavir was observed with the screening variants and F1, with each displaying similar FC EC_50_ values of up to 11- and 8-fold respectively, as shown in Figure 2(c). Screening variants remained susceptible to darunavir, although variant F4 displayed a 5-fold reduction in PI susceptibility. Use of EC_90_ did not significantly change the relationships between clones (Figure S1). MPWGD was reduced at the time of treatment failure (0.002) in comparison with baseline (0.009, *P* < 0.0001). Screening and failure viral variants clustered in a stepwise fashion in a maximum likelihood analysis (Figure 3), consistent with selective pressure and ongoing viral evolution between the two timepoints.

The second individual (Patient 4201) was infected with a subtype G virus and experienced virological failure at week 48 of the trial. The patient had a lopinavir trough concentration >6000 ng/mL at this timepoint, though levels were undetectable at the week 24 visit. Adherence reporting suggested erratic drug exposure from week 20 onwards. A number of protease polymorphisms were present both at baseline and time of treatment failure: I13V, K14R, K20I, E35Q, M36I, R41K, R57K, Q61N, C67E, H69K, V82I and L89M (Table S1). In addition, a number of mutations that have been previously associated with PI susceptibility or resistance in Gag were present at both screening and time of treatment failure: E12K, R76K, H219Q, V370A, R380K, K436R and S451N (Table S2).

Five variants were subjected to phenotypic analysis: four derived from baseline (S1, S3, S4 and S5) and one from failure (F2). The baseline viral variants S1 and S5 displayed significant reductions in susceptibility to a number of PIs, most notably to lopinavir where ≥16-fold reduction in susceptibility in comparison with the reference strain was observed (Figure 2d). Reduced susceptibility to atazanavir (up to 11-fold), darunavir (up to 5-fold) and saquinavir (up to 4-fold) were also observed. This reduction in susceptibility was also observed for the viral variant derived from the failure timepoint, F2. The other variants derived from the baseline timepoint, S3 and S4, exhibited less pronounced reductions in susceptibility to lopinavir of 8- and 11-fold, respectively. In contrast to the other patients studied, the FC for Patient 4201 remained >10, even when considering EC_90_ (Figure S1). Furthermore, the use of EC_90_ revealed two screening variants with >5 FC to darunavir, which were <5-fold when EC_50_ was considered. Finally, there was a significant difference in MPWGD between baseline (0.014) and time of treatment failure (0.001, *P* < 0.0001). The screening and failure viral variants clustered separately on the maximum likelihood phylogenetic tree, supporting ongoing viral evolution during PI monotherapy (Figure 3).

### Virological failure with ‘wild-type’ virus after prolonged suppression

Patient 508 was infected with subtype B and experienced late therapy failure at week 96 with a viral load at 25 300 copies/mL. Although lopinavir plasma trough concentrations were unavailable beyond week 48, those prior to this timepoint were consistently >2000 ng/mL and very few missed doses were reported. A number of protease polymorphisms were present both at baseline and time of treatment failure: N37S, P39Q, I62V, L63P, V77I and I93L. Only one change in Gag associated with PI susceptibility or resistance was present—V370A (V/A mixture pre-therapy and all V370A at failure, Tables S1 and S2).

Phenotypic susceptibility was measured for six variants in total: three each from baseline (S1, S2 and S3) and the time of treatment failure (F2, F3 and F9). Figure 2(e) shows that for lopinavir, most viral variants remained susceptible with up to 4-fold reduction in susceptibility in comparison with the reference strain. In addition, viral variants were susceptible to darunavir. Use of EC_90_ instead of EC_50_ did not significantly change the relationships between clones (Figure S1). MPWGD at baseline (0.011) and time of treatment failure (0.010, *P* = 0.07) were not significantly different and baseline and failure viral variants intermingled on phylogenetic analysis, in contrast to the other patients studied (Figure 3). This is consistent with a dramatic change in adherence after a prolonged period of suppression. Of note, bootstrap values were relatively low for the nodes between the branches within each patient, likely due to the close relatedness of the variants.

## Discussion

The MONARK clinical trial offered the unique opportunity to carry out the most detailed analysis of PI/r monotherapy failure to date, focusing on the most relevant patients failing lopinavir/ritonavir single-drug first-line treatment: those who do so in the absence of major PI mutations by bulk population sequencing. Given the evidence for the importance of the inclusion of full-length *gag* alongside its coevolved protease in phenotypic assays, we set out to examine in this proof-of-concept study whether testing phenotypic PI susceptibility of multiple full-length *gag–protease* sequences from individual patients failing therapy in the absence of major PI resistance mutations could be more illuminating. The paired nature of these samples, one taken before treatment and one at time of treatment failure, enabled a comparison of PI susceptibility and amino acid sequence of viruses before and after PI/r monotherapy. Uniquely, we were able to contextualize our phenotypic data with lopinavir plasma concentrations, adherence and clinical data.

We show using multiple patient-derived *gag–protease* sequences that decreases in phenotypic susceptibility can occur (in two of four failures occurring before 96 weeks) when no major PI mutations during lopinavir/ritonavir therapy are detected using standard techniques. Use of multiple clones in phenotypic analysis enabled us to test lower-frequency variants and this approach was vindicated by the detection of a protease V82A major mutation in a single clone at virological failure in Patient 1403. This viral population with reduced lopinavir susceptibility (17-fold for lopinavir) would have been missed by both consensus sequencing and consensus strain susceptibility testing. We detected a second individual (Patient 3204) with an emergent reduction in PI susceptibility (13-fold to lopinavir), also on a background of reduced susceptibility and associated with amino acid changes in Gag and M36I in protease. M36I has been associated with PI exposure,^26,36^ though was not linked to reduced susceptibility in a commercial phenotypic susceptibility system incorporating only patient-derived *protease* sequences.^37^ The resistance pathway involving M36I could therefore involve the Gag positions in Table S2.

The lower and upper clinical cut-offs (CCOs) for lopinavir are 10 and 50, derived using a protease-containing phenotypic assay with an assay reference strain also derived from NL4.3.^38^ The CCOs for lopinavir/ritonavir monotherapy have not been established but may be lower given that CCOs are derived from clinical studies conducted with triple-agent antiretroviral therapy. In light of these data, one would not restart lopinavir/ritonavir monotherapy in these individuals and atazanavir is probably not an alternative given the overlapping susceptibility profiles observed both in the presence^39^ and absence of major protease mutations.^25,26^

An alternative to restarting the same PI in resource-limited settings might be a switch to a darunavir/ritonavir-based third-line regimen. However, this study has revealed a possible compromise of darunavir activity in three of five individuals with virological failure. In Patient 4201, there was an FC of 5 relative to the reference strain both before and after virological failure. Comparable levels of reduction in darunavir susceptibility were previously seen in only 1 of 15 treatment-naive isolates tested using this assay (9 subtype B,^26^ 3 subtype A and 3 subtype C^25^). Reduced darunavir susceptibility of 5-fold emerged at failure in Patients 3204 and 1404, having been significantly lower pre-therapy (Figure 2). Although the darunavir CCO has been set at 10-fold (in a commercial protease-only vector system), this might be too high in settings of darunavir monotherapy or functional monotherapy. Further studies are needed to properly define CCOs in the context of a *gag–protease* phenotypic assay and also for PI monotherapy. Notably, the darunavir susceptibility score is based around protease mutations (as are all scoring systems) and would not have detected the reduced susceptibility observed in our study. Therefore, our approach, if validated by larger studies, could potentially be used to assess patients for darunavir/ritonavir-based salvage therapy.

Finally, our work has demonstrated some degree of reduced baseline susceptibility (>5-fold to any PI) in all five patients. Data for one patient in particular, Patient 4201, provided evidence that a significant reduction in PI susceptibility at baseline may contribute to treatment failure. The baseline variants for this patient displayed up to 16-fold reduction in susceptibility to lopinavir (using both EC_50_ and EC_90_) that was also present at treatment failure. This degree of reduction in PI susceptibility was the same as observed for variant 1403 F6, which contained the V82A major PI resistance mutation, implying clinical relevance in the FCs observed for *gag*-associated mutations. Patient 4201 failed at week 48 of the clinical trial, at which time an adequate lopinavir trough plasma concentration was present and self-reported adherence was high at most appointments. This indicates that the reduced PI susceptibility may have enabled viral replication in the presence of PI/r monotherapy. We hypothesize that the reduced PI susceptibility present in baseline viral variants reduces the therapeutic forgiveness of suboptimal adherence, thus rendering these patients more likely to experience virological failure.

Of note, our findings are in patients treated with lopinavir/ritonavir monotherapy and therefore may not be generalizable to triple-therapy regimens containing this PI. Furthermore, treatment guidelines now specify that boosted lopinavir or darunavir should only be used as monotherapy following viral suppression with a triple combination regimen (http://www.eacsociety.org/Portals/0/140601_EACS%20EN7.02.pdf). The characteristics of emerging virus populations following maintenance may not be the same as those observed in our analysis. A final consideration relates to data from both MONET and OK04 demonstrating successful resuppression with the addition of two nucleosides following viral rebound on maintenance with PI monotherapy.^16,17^ In the latter, resistance testing was undertaken and two nucleosides only reintroduced if there was no genotypic evidence of lopinavir resistance.^16^ It is still possible that the period of suppression under maintenance strategies will differentially impact the nature of emerging virus at rebound. Our approach should therefore be repeated using samples from maintenance studies in the future.

Several limitations to this study deserve mention. We have used a bulk PCR approach that can be prone to recombination. The alternative, single genome amplification, could be biased by the fact that one would need to select ‘representative’ sequences from >30 sequences for phenotyping. Secondly, the sample size is relatively small, despite the intensive clonal analyses at baseline and failure for each patient. Thirdly, our vector system does not incorporate native envelope, proposed as being a determinant of PI susceptibility whilst this manuscript was in preparation.^40^ Finally, use of a single reference strain may be a limitation, although all viruses were compared with the same reference strain in this study as in previous publications.^25–27^ Future studies will be needed to establish the utility of coevolved *gag–pro–env* in the assessment of drug susceptibility in clinical isolates and to further address differential susceptibility to PI in determining clinical outcomes.

### Conclusions

This study represents the most comprehensive assessment of PI susceptibility to date. It highlights the complex nature of virological failure to this class of antiretroviral, suggesting baseline reduced susceptibility, evolution of resistance or a mixture of both can be contributing factors. Use of a phenotyping approach also reveals lower darunavir susceptibility, warranting further study as this agent is commonly used following lopinavir failure.

## Funding

This study was funded by the Wellcome Trust and supported by a grant from AbbVie Laboratories. R. K. G. is funded by a Wellcome Trust Fellowship (WT093722MA). K. A. S. was funded by Public Health England (formerly the Health Protection Agency).

## Transparency declarations

I. C.-C. is an employee of AbbVie Laboratories and may own stock or options in AbbVie. I. C.-C. was involved in interpretation of data and review of the manuscript; however, final content was decided by the principal investigator (R. K. G.). All other authors: none to declare.

## Supplementary data

Table S1, Table S2 and Figure S1 are available as Supplementary data at *JAC* Online (http://jac.oxfordjournals.org/).

Supplementary Data

## References

[1] Hosseinipour MC, van Oosterhout JJ, Weigel R (2009). The public health approach to identify antiretroviral therapy failure: high-level nucleoside reverse transcriptase inhibitor resistance among Malawians failing first-line antiretroviral therapy. AIDS.

[2] Gupta RK, Hill A, Sawyer AW (2009). Virological monitoring and resistance to first-line highly active antiretroviral therapy in adults infected with HIV-1 treated under WHO guidelines: a systematic review and meta-analysis. Lancet Infect Dis.

[3] Sungkanuparph S, Manosuthi W, Kiertiburanakul S (2007). Options for a second-line antiretroviral regimen for HIV type 1-infected patients whose initial regimen of a fixed-dose combination of stavudine, lamivudine, and nevirapine fails. Clin Infect Dis.

[4] Hosseinipour MC, Gupta RK, Van Zyl G (2013). Emergence of HIV drug resistance during first- and second-line antiretroviral therapy in resource-limited settings. J Infect Dis.

[5] Messou E, Chaix ML, Gabillard D (2013). Increasing rate of TAMs and etravirine resistance in HIV-1-infected adults between 12 and 24 months of treatment: the VOLTART cohort study in Cote d'Ivoire, West Africa. J Acquir Immune Defic Syndr.

[6] Murphy RA, Sunpath H, Castilla C (2012). Second-line antiretroviral therapy: long-term outcomes in South Africa. J Acquir Immune Defic Syndr.

[7] Ajose O, Mookerjee S, Mills EJ (2012). Treatment outcomes of patients on second-line antiretroviral therapy in resource-limited settings: a systematic review and meta-analysis. AIDS.

[8] Saravanan S, Vidya M, Balakrishnan P (2012). Viremia and HIV-1 drug resistance mutations among patients receiving second-line highly active antiretroviral therapy in Chennai, Southern India. Clin Infect Dis.

[9] Gilks CF, Walker AS, Dunn DT (2012). Lopinavir/ritonavir monotherapy after 24 weeks of second-line antiretroviral therapy in Africa: a randomized controlled trial (SARA). Antivir Ther.

[10] Paton NI, Kityo C, Hoppe A (2014). Assessment of second-line antiretroviral regimens for HIV therapy in Africa. N Engl J Med.

[11] Bunupuradah T, Chetchotisakd P, Ananworanich J (2012). A randomized comparison of second-line lopinavir/ritonavir monotherapy versus tenofovir/lamivudine/lopinavir/ritonavir in patients failing NNRTI regimens: the HIV STAR study. Antivir Ther.

[12] Bartlett JA, Ribaudo HJ, Wallis CL (2012). Lopinavir/ritonavir monotherapy after virologic failure of first-line antiretroviral therapy in resource-limited settings. AIDS.

[13] Ghosn J, Flandre P, Cohen-Codar I (2010). Long-term (96-week) follow-up of antiretroviral-naive HIV-infected patients treated with first-line lopinavir/ritonavir monotherapy in the MONARK trial. HIV Med.

[14] Delfraissy JF, Flandre P, Delaugerre C (2008). Lopinavir/ritonavir monotherapy or plus zidovudine and lamivudine in antiretroviral-naive HIV-infected patients. AIDS.

[15] Arribas JR, Clumeck N, Nelson M (2012). The MONET trial: week 144 analysis of the efficacy of darunavir/ritonavir (DRV/r) monotherapy versus DRV/r plus two nucleoside reverse transcriptase inhibitors, for patients with viral load <50 HIV-1 RNA copies/mL at baseline. HIV Med.

[16] Pulido F, Arribas JR, Delgado R (2008). Lopinavir-ritonavir monotherapy versus lopinavir-ritonavir and two nucleosides for maintenance therapy of HIV. AIDS.

[17] Arribas JR, Delgado R, Arranz A (2009). Lopinavir-ritonavir monotherapy versus lopinavir-ritonavir and 2 nucleosides for maintenance therapy of HIV: 96-week analysis. J Acquir Immune Defic Syndr.

[18] Delaugerre C, Flandre P, Chaix ML (2009). Protease inhibitor resistance analysis in the MONARK trial comparing first-line lopinavir-ritonavir monotherapy to lopinavir-ritonavir plus zidovudine and lamivudine triple therapy. Antimicrob Agents Chemother.

[19] Hill A, McBride A, Sawyer AW (2013). Resistance at virological failure using boosted protease inhibitors versus nonnucleoside reverse transcriptase inhibitors as first-line antiretroviral therapy: implications for sustained efficacy of ART in resource-limited settings. J Infect Dis.

[20] Gupta R, Hill A, Sawyer AW (2008). Emergence of drug resistance in HIV type 1-infected patients after receipt of first-line highly active antiretroviral therapy: a systematic review of clinical trials. Clin Infect Dis.

[21] Ghosn J, Delaugerre C, Flandre P (2011). Polymorphism in Gag gene cleavage sites of HIV-1 non-B subtype and virological outcome of a first-line lopinavir/ritonavir single drug regimen. PLoS One.

[22] Fun A, Wensing AM, Verheyen J (2012). Human immunodeficiency virus Gag and protease: partners in resistance. Retrovirology.

[23] McKinnon JE, Delgado R, Pulido F (2011). Single genome sequencing of HIV-1 gag and protease resistance mutations at virologic failure during the OK04 trial of simplified versus standard maintenance therapy. Antivir Ther.

[24] Ho SK, Perez EE, Rose SL (2009). Genetic determinants in HIV-1 Gag and Env V3 are related to viral response to combination antiretroviral therapy with a protease inhibitor. AIDS.

[25] Gupta RK, Kohli A, McCormick AL (2010). Full-length HIV-1 Gag determines protease inhibitor susceptibility within in vitro assays. AIDS.

[26] Sutherland KA, Mbisa JL, Cane PA (2014). Contribution of Gag and protease to variation in susceptibility to protease inhibitors between different strains of subtype B HIV-1. J Gen Virol.

[27] Parry CM, Kohli A, Boinett CJ (2009). Gag determinants of fitness and drug susceptibility in protease inhibitor-resistant human immunodeficiency virus type 1. J Virol.

[28] Van Laethem K, Schrooten Y, Dedecker S (2006). A genotypic assay for the amplification and sequencing of gag and protease from diverse human immunodeficiency virus type 1 group M subtypes. J Virol Methods.

[29] Parry CM, Kolli M, Myers RE (2011). Three residues in HIV-1 matrix contribute to protease inhibitor susceptibility and replication capacity. Antimicrob Agents Chemother.

[30] Tamura K, Peterson D, Peterson N (2011). MEGA5: molecular evolutionary genetics analysis using maximum likelihood, evolutionary distance, and maximum parsimony methods. Mol Biol Evol.

[31] Guindon S, Dufayard JF, Lefort V (2010). New algorithms and methods to estimate maximum-likelihood phylogenies: assessing the performance of PhyML 3.0. Syst Biol.

[32] de Oliveira T, Deforche K, Cassol S (2005). An automated genotyping system for analysis of HIV-1 and other microbial sequences. Bioinformatics.

[33] Alcantara LC, Cassol S, Libin P (2009). A standardized framework for accurate, high-throughput genotyping of recombinant and non-recombinant viral sequences. Nucl Acids Res.

[34] Aoki M, Venzon DJ, Koh Y (2009). Non-cleavage site gag mutations in amprenavir-resistant human immunodeficiency virus type 1 (HIV-1) predispose HIV-1 to rapid acquisition of amprenavir resistance but delay development of resistance to other protease inhibitors. J Virol.

[35] King MS, Rode R, Cohen-Codar I (2007). Predictive genotypic algorithm for virologic response to lopinavir-ritonavir in protease inhibitor-experienced patients. Antimicrob Agents Chemother.

[36] Santos JR, Llibre JM, Imaz A (2012). Mutations in the protease gene associated with virological failure to lopinavir/ritonavir-containing regimens. J Antimicrob Chemother.

[37] Kempf DJ, King MS, Bernstein B (2004). Incidence of resistance in a double-blind study comparing lopinavir/ritonavir plus stavudine and lamivudine to nelfinavir plus stavudine and lamivudine. J Infect Dis.

[38] Kempf DJ, Isaacson JD, King MS (2001). Identification of genotypic changes in human immunodeficiency virus protease that correlate with reduced susceptibility to the protease inhibitor lopinavir among viral isolates from protease inhibitor-experienced patients. J Virol.

[39] Johnson VA, Calvez V, Gunthard HF Update of the Drug Resistance Mutations in HIV-1: March 2013. http://www.iasusa.org/sites/default/files/tam/21-1-6.pdf.

[40] Rabi SA, Laird GM, Durand CM (2013). Multi-step inhibition explains HIV-1 protease inhibitor pharmacodynamics and resistance. J Clin Invest.

