# Supplementary material for: Phenotypic characterization of virological failure following lopinavir/ritonavir monotherapy using full-length gag–protease genes
Source: J Antimicrob Chemother. 2014 Aug 4;69(12):3340–8. doi: 10.1093/jac/dku296 (PMC4228778; doi:10.1093/jac/dku296)
Supplement: Supplementary Data [file supp_69_12_3340__index.html]

Phenotypic characterization of virological failure following lopinavir/ritonavir monotherapy using full-length gag–protease genes — Phenotypic characterization of virological failure following lopinavir/ritonavir monotherapy using full-length gag–protease genes — Supplementary Data 

# Phenotypic characterization of virological failure following lopinavir/ritonavir monotherapy using full-length *gag–protease* genes

## Supplementary Data

Supplementary Data

**Files in this Data Supplement:**

- Supplementary Data - Docx file
