# Supplementary material for: Phenotypic characterization of virological failure following lopinavir/ritonavir monotherapy using full-length gag–protease genes
Source: J Antimicrob Chemother. 2014 Aug 4;69(12):3340–8. doi: 10.1093/jac/dku296 (PMC4228778; doi:10.1093/jac/dku296)

| **Patient** | **Protease Inhibitor Resistance mutations*** | | **Protease polymorphisms** |
| --- | --- | --- | --- |
|  | Major | Minor |  |
| 1403 | V82A  (F6) |  | I13V, K14R, G16E, K20I, E35D, M36I, H69K, K70R, L89M |
| 3204 |  |  | E35D, M36I (F1-10), N37S (S1-9), L63P, I72V, V77I, I93L |
| 1404 |  | A71T | I13A, K20I, M36I, R41K, I64V (S1,2,3,4,6,7,9), H69K, I72M (F1-10), L89I, I93M |
| 4201 |  |  | I13V, K14R, K20I, E35Q, M36I, R41K, G52R (S8), R57K, Q61N, Q61D (S7), I64M (S1,2,3,4,5,6,8,9,10), C67E, H69K, I72V (S2,4,6), V82I, L89M |
| 508 |  |  | T31I (F1), N37S, P39Q, S47N (S2), Q49L (S5), I62V, L63P, V77I, I93L |

**Supplementary data**

**Table S1.** **Protease mutations in each patient and classification into major, minor and polymorphic changes**.

*Amino acid changes were in all viral variants except where indicated.

F=fail variant, S=screen variant.

Generated using the Stanford resistance database algorithm: <http://hivdb.stanford.edu>

**Table S2. Previously reported Gag changes in viral variants**

| **Reported Mutation** | **Viral variants from patient #1403** | | | | | | | | | | | | | | | | | | | |
| --- | --- | --- | --- | --- | --- | --- | --- | --- | --- | --- | --- | --- | --- | --- | --- | --- | --- | --- | --- | --- |
|  | **Screening** | | | | | | | | | | **Failure** | | | | | | | | | |
|  | **1** | **2** | **3** | **4** | **5** | **6** | **7** | **8** | **9** | **10** | **1** | **2** | **3** | **4** | **5** | **6** | **7** | **8** | **9** | **10** |
| **E12K** | K | K | K | K | K | K | K | K | K | K | K | K | K | K | K | K | K | K | K | K |
| **R76K** | K | K | K | K | K | K | K | K | K | K | K | K | K | K | K | K | K | K | K | K |
| **Y79F** | Y | Y | Y | Y | Y | Y | Y | Y | Y | Y | Y | Y | Y | Y | Y | F | Y | Y | F | Y |
| **T375N** | N | N | N | N | N | N | N | N | N | N | N | N | N | N | N | N | N | N | N | N |
| **I376V** | V | V | V | V | V | V | V | V | V | V | I | I | V | V | V | I | V | V | V | V |
| **L449F/P** | P | P | P | P | P | P | P | P | P | P | P | P | P | P | P | P | P | P | P | P |

| **Reported Mutation** | **Viral variants from patient #3204** | | | | | | | | | | | | | | | | | | | |
| --- | --- | --- | --- | --- | --- | --- | --- | --- | --- | --- | --- | --- | --- | --- | --- | --- | --- | --- | --- | --- |
|  | **Screening** | | | | | | | | | | **Failure** | | | | | | | | | |
|  | **1** | **2** | **3** | **4** | **5** | **6** | **7** | **8** | **9** | **10** | **1** | **2** | **3** | **4** | **5** | **6** | **7** | **8** | **9** | **10** |
| **E12K** | K | Q | K | K | K | Q | Q | K | K | K | K | Q | K | K | K | K | K | K | K | K |
| **R76K** | R | R | R | R | K | R | R | R | R | R | R | R | R | R | R | R | R | R | R | R |
| **Y79F** | F | F | F | F | F | F | F | F | F | F | F | F | F | F | F | F | F | F | F | F |
| **H219Q/P** | Q | Q | Q | Q | Q | Q | Q | Q | Q | Q | Q | Q | Q | Q | Q | Q | Q | Q | Q | Q |
| **T81A** | A | T | T | T | T | T | T | T | T | T | A | A | A | A | A | A | A | A | A | A |
| **M200I** | I | I | I | I | I | I | I | I | I | I | I | I | I | I | I | I | I | I | I | I |
| **H219Q** | Q | Q | Q | Q | Q | Q | Q | Q | Q | Q | Q | Q | Q | Q | Q | Q | Q | Q | Q | K |
| **S373P** | P | P | S | S | P | S | P | S | P | S | S | P | S | S | S | S | S | S | S | S |
| **T375N/S** | N | N | N | N | N | N | N | N | N | N | N | N | N | N | N | N | N | N | N | N |
| **I376V** | I | I | V | V | I | I | I | V | I | V | I | I | I | I | I | I | I | I | I | I |
| **R380K** | K | K | K | K | K | K | K | K | K | K | K | K | K | K | K | K | K | K | K | K |
| **I389T** | M | M | T | T | M | T | M | T | M | T | T | M | T | T | T | T | T | T | T | T |
| **S451N** | S | N | S | S | N | S | N | S | S | N | S | S | S | S | S | S | S | S | S | S |

| **Reported Mutation** | **Viral variants from patient #1404** | | | | | | | | | | | | | | | | | | | |
| --- | --- | --- | --- | --- | --- | --- | --- | --- | --- | --- | --- | --- | --- | --- | --- | --- | --- | --- | --- | --- |
|  | **Screening** | | | | | | | | | | **Failure** | | | | | | | | | |
|  | **1** | **2** | **3** | **4** | **5** | **6** | **7** | **8** | **9** | **10** | **1** | **2** | **3** | **4** | **5** | **6** | **7** | **8** | **9** | **10** |
| **E12K** | K | K | K | K | K | K | K | K | K | K | K | K | K | K | K | K | K | K | K | K |
| **R76K** | K | K | K | K | K | K | K | K | K | K | K | K | K | K | K | K | K | K | K | K |
| **Y79F** | Y | Y | Y | F | Y | Y | Y | Y | Y | Y | Y | Y | Y | Y | Y | Y | Y | Y | Y | Y |
| **V370A/M** | A | A | A | A | A | A | A | A | A | A | A | A | A | A | A | A | A | A | A | A |
| **L449F/P** | P | P | P | P | P | P | P | P | P | P | P | P | P | P | P | P | P | P | P | P |

| **Reported Mutation** | **Viral variants from patient #4201** | | | | | | | | | | | | | | | | | | | |
| --- | --- | --- | --- | --- | --- | --- | --- | --- | --- | --- | --- | --- | --- | --- | --- | --- | --- | --- | --- | --- |
|  | **Screening** | | | | | | | | | | **Failure** | | | | | | | | | |
|  | **1** | **2** | **3** | **4** | **5** | **6** | **7** | **8** | **9** | **10** | **1** | **2** | **3** | **4** | **5** | **6** | **7** | **8** | **9** | **10** |
| **E12K** | K | K | K | K | K | K | K | K | K | K | K | K | K | K | K | K | K | K | K | K |
| **R76K** | K | K | K | K | K | K | K | K | K | K | K | K | K | K | K | K | K | K | K | K |
| **Y79F** | Y | F | F | F | F | F | F | F | F | Y | Y | Y | Y | Y | Y | Y | Y | Y | Y | Y |
| **H219Q/P** | Q | Q | Q | Q | Q | Q | Q | Q | Q | Q | Q | Q | Q | Q | Q | Q | Q | Q | Q | Q |
| **V370A/M** | A | A | A | A | A | A | A | A | A | A | A | A | A | A | A | A | A | A | A | A |
| **R380K** | K | K | K | K | K | K | K | K | K | K | K | K | K | K | K | K | K | K | K | K |
| **K436E/R** | R | R | R | R | R | R | R | R | R | R | R | R | R | R | R | R | R | R | R | R |
| **S451N** | N | N | N | N | N | N | N | N | N | N | N | N | N | N | N | N | N | N | N | N |

| **Reported Mutation** | **Viral variants from patient #508** | | | | | | | | | | | | | | | | | | | |
| --- | --- | --- | --- | --- | --- | --- | --- | --- | --- | --- | --- | --- | --- | --- | --- | --- | --- | --- | --- | --- |
|  | **Screening** | | | | | | | | | | **Failure** | | | | | | | | | |
|  | **1** | **2** | **3** | **4** | **5** | **6** | **7** | **8** | **9** | **10** | **1** | **2** | **3** | **4** | **5** | **6** | **7** | **8** | **9** | **10** |
| **V128A** | V | V | V | V | V | V | A | V | V | V | V | V | V | V | V | V | V | V | V | V |
| **V370A/M** | A | V | A | A | A | A | A | V | A | A | A | A | A | A | A | A | A | A | A | A |
| **R380K** | R | R | R | R | R | R | R | R | R | R | R | K | R | R | R | R | R | R | R | R |
| **S451N** | S | S | S | S | S | S | S | S | S | S | S | S | N | S | S | S | S | S | S | S |


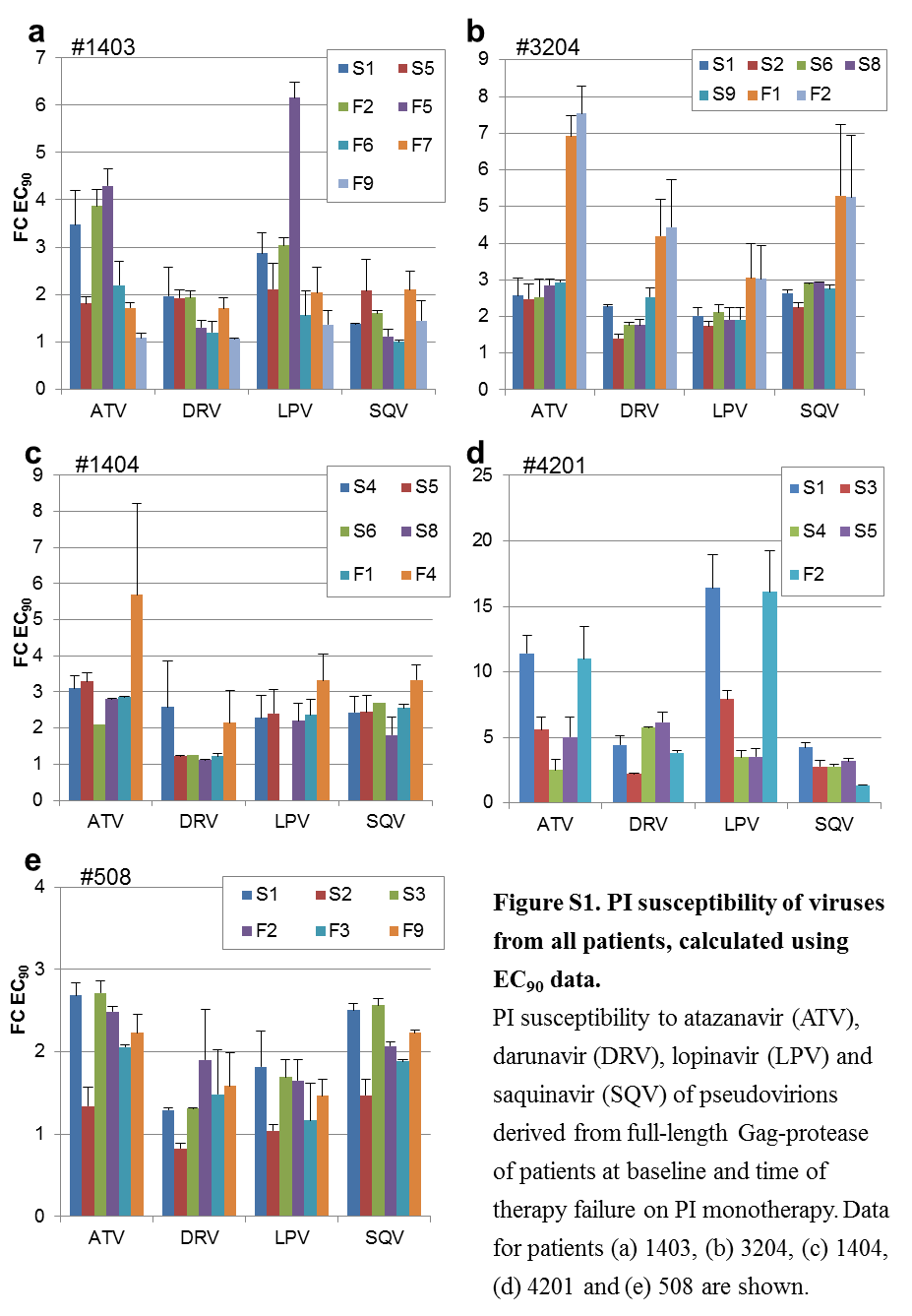

Supplement: Supplementary Data [file supp_dku296_dku296supp.docx]
